# Past dynamics of HIV transmission among men who have sex with men in Montréal, Canada: a mathematical modeling study

**DOI:** 10.1186/s12879-022-07207-7

**Published:** 2022-03-07

**Authors:** Rachael M. Milwid, Yiqing Xia, Carla M. Doyle, Joseph Cox, Gilles Lambert, Réjean Thomas, Sharmistha Mishra, Daniel Grace, Nathan J. Lachowsky, Trevor A. Hart, Marie-Claude Boily, Mathieu Maheu-Giroux

**Affiliations:** 1 Department of Epidemiology and Biostatistics, School of Population and Global Health, McGill University, Montréal, QC Canada; 2 Direction Régionale de Santé Publique de Montréal, Montréal, QC Canada; 3 Clinique Médicale L’Actuel, Montréal, QC Canada; 4 Institute of Health Policy, Management and Evaluation, University of Toronto, Toronto, ON Canada; 5 Social and Behavioural Health Sciences, Dalla Lana School of Public Health, University of Toronto, Toronto, ON Canada; 6 School of Public Health and Social Policy, University of Victoria, Victoria, BC Canada; 7 Department of Psychology, Ryerson University, Toronto, ON Canada; 8 Department of Infectious Diseases, Imperial College London, London, UK

**Keywords:** HIV/AIDS, Gay bisexual and other men who have sex with men (gbMSM), Mathematical modeling, Agent-based model, Approximate Bayesian Computation Sequential Monte Carlo, Treatment and care cascade

## Abstract

**Background:**

Gay, bisexual, and other men who have sex with men (gbMSM) experience disproportionate risks of HIV acquisition and transmission. In 2017, Montréal became the first Canadian Fast-Track City, setting the 2030 goal of zero new HIV infections. To inform local elimination efforts, we estimate the evolving role of prevention and sexual behaviours on HIV transmission dynamics among gbMSM in Montréal between 1975 and 2019.

**Methods:**

Data from local bio-behavioural surveys were analyzed to develop, parameterize, and calibrate an agent-based model of sexual HIV transmission. Partnership dynamics, HIV’s natural history, and treatment and prevention strategies were considered. The model simulations were analyzed to estimate the fraction of HIV acquisitions and transmissions attributable to specific groups, with a focus on age, sexual partnering level, and gaps in the HIV care-continuum.

**Results:**

The model-estimated HIV incidence peaked in 1985 (2.3 per 100 person years (PY); 90% CrI: 1.4–2.9 per 100 PY) and decreased to 0.1 per 100 PY (90% CrI: 0.04–0.3 per 100 PY) in 2019. Between 2000–2017, the majority of HIV acquisitions and transmissions occurred among men aged 25–44 years, and men aged 35–44 thereafter. The unmet prevention needs of men with > 10 annual anal sex partners contributed 90–93% of transmissions and 67–73% of acquisitions annually. The primary stage of HIV played an increasing role over time, contributing to 11–22% of annual transmissions over 2000–2019. In 2019, approximately 70% of transmission events occurred from men who had discontinued, or never initiated antiretroviral therapy.

**Conclusions:**

The evolving HIV landscape has contributed to the declining HIV incidence among gbMSM in Montréal. The shifting dynamics identified in this study highlight the need for continued population-level surveillance to identify gaps in the HIV care continuum and core groups on which to prioritize elimination efforts.

**Supplementary Information:**

The online version contains supplementary material available at 10.1186/s12879-022-07207-7.

## Introduction

From the beginning of the HIV epidemic, gay, bisexual, and other men who have sex with men (gbMSM) have been disproportionately impacted [1]. In 2019, 60% of new diagnoses in Québec were recorded in Montréal, Canada’s second most populated city [2]. Of the new diagnoses, 70% occurred among men, of which 71% were reported among gbMSM [3].

In 2017, Montréal became the first Canadian *Fast-Track City*, pledging to reach zero new HIV infections by 2030 [4]. The successful achievement of this ambitious goal largely depends on the implementation of effective city-level public health initiatives to reduce the acquisition and transmission potential for HIV. Understanding the impact of sexual behaviors and specific HIV interventions on the epidemic can aid in the development of relevant HIV elimination plans [5]. This objective is complicated, however, by the continued evolution of the complex HIV prevention and treatment landscape, which includes: HIV testing (1985) [6], anti retroviral therapy (ART; 1996) [7], post-exposure prophylaxis (PEP; 2001) [8], and pre-exposure prophylaxis (PrEP; 2013) [8].

The scarcity of gbMSM survey data from the early years of the epidemic adds to the challenges of characterizing HIV epidemics and their complex transmission dynamics using traditional epidemiological methods. Tools such as agent-based models (ABM) –computer simulations of epidemics– are appropriate to understand and characterize past and future transmission dynamics [9–12]. Mathematical models of HIV transmission have been used to describe the drivers (e.g., age, infection stage, knowledge of status, and treatment status [14, 15]) and dynamics of global and local HIV epidemics. These models have focused on different population subgroups, have been applied to a range of geographical locations, and have studied, both prospectively and retrospectively, the impact of various interventions on HIV dynamics [11, 16–18]. The relevance and applicability of the models is largely dependent on the quality and quantity of the data used to parameterize and inform the population-specific model [19].

Despite the quantity of available Montréal-based sexual behavioral data, there have been no recent attempts to systematically analyze and triangulate data and surveys on HIV transmission among gbMSM in Montréal to understand evolving transmission dynamics. The purpose of this study was to describe the epidemiological characteristics of the HIV epidemic and understand the sources of HIV acquisition and transmission between 1975 and 2019.

## Methods

### Data analysis

Data from the peer-reviewed and grey literature, and individual-level data from the two cross-sectional *Argus* surveys (2005; n = 1957 and 2008; n = 1873), the *Engage* cohort (2017; n = 1179, 2018; n = 887, 2019; n = 850), and the *L’Actuel PrEP Cohort* (2013–2019; n = 2746) studies were analyzed to identify temporal trends in sexual behaviors and HIV interventions uptake. Details on the studies can be found elsewhere [20–26]. Briefly, the *Argus* surveys used venue-based convenience sampling to recruit gbMSM aged 18 + years from the island of Montréal who reported sex with another man during their lifetime. The longitudinal *Engage* cohort used respondent-driven sampling to recruit gbMSM aged 16 + years from Montréal who reported sex with another man within the past six months. The *Argus* data were standardized (by age and ethnicity) to the *Engage* data which were analyzed using Volz-Heckathorn weights [27]. The data analysis was used to inform model development, parameterization, and calibration (see Additional file 1).

### Agent-based model

An ABM was developed to simulate in discrete time, the sexual transmission dynamics of HIV among gbMSM aged 15 + years in Montréal. The model represents an open population, matching the demographic and epidemiological characteristics reported in the surveys, health, and census data. A detailed model description can be found in the Additional file 1. The main characteristics of the partnership formation, natural disease progression, and HIV interventions are described below. A complete list of model parameters (demographic, sexual behaviors, biological, and interventions) is presented in the Additional file 1.

#### Partnership formation and duration

Upon entry into the population, men are assigned an annual partner change rate, informed by the survey data. Only partnerships in which anal sex is performed are modelled. As individuals age, their partner change rate increases slightly up to age 30 and then decreases, in line with the survey data. In order to parameterize intervention uptake and estimate epidemic drivers, men having  ≤ 5, 6–10, and > 10 anal sex partners per year are categorized as being in the low, medium, and high sexual partnering groups, respectively [28]. Men are assigned a partner based on data-driven mixing matrices that vary by age, reported serostatus, and preferred anal sex role (insertive, receptive, or versatile). If no available partners match all three criteria, the search continues with either age or serostatus dropped. If there are still no suitable partners, the man does not form a partnership at that time.

Men can form casual or regular partnerships. Casual partnerships begin and end within the same two-week time step (but can involve more than one sex act), while regular partnerships are assumed to exceed two or more time steps in duration. Men can only have one concurrent regular partnership. Therefore, upon partnership formation, if either man is already part of a regular partnership, then the partnership is assigned a casual status. Otherwise, a Bernoulli distribution is used to determine the partnership type.

#### HIV transmission and progression

It is assumed that there are no external sources of sexual HIV transmission (e.g., in the context of sex with female partners or sharing injecting needles). Following HIV acquisition, individuals go through an initial short, highly infectious period [29]. Subsequently, people living with HIV (PLHIV) progress through distinct CD4 cell count categories, with some starting at a lower CD4 cell count [30]: > 500 cells $\mu {L}^{-1}$, 350–500 cells $\mu {L}^{-1}$, 200–350 cells $\mu {L}^{-1}$, and < 200 cells $\mu {L}^{-1}$. Men on ART maintain their CD4 cell count from treatment initiation until ART discontinuation or death [31]. The AIDS-related death rate varies by age, stage of infection, treatment status, and time on ART [32, 33].

The probability of HIV transmission within sero-discordant partnerships is computed as a function of the number of sex acts in the partnership, the HIV-positive partner’s stage of infection and ART status, and the HIV-negative partner’s sex role, PrEP use, the proportion of sex acts protected by condoms within the partnership, and whether the man seeks PEP afterward.

#### HIV interventions

All men have similar HIV testing rates prior to 1996. Subsequently, the annual rates of HIV testing were estimated from survey data and were allowed to vary by sexual partnering levels (low versus moderate/high). Once tested for HIV, a minimum inter-testing interval is assigned. From 1985 onwards, testing occurs for undiagnosed symptomatic PLHIV on average six months after progressing to the late stage of infection, irrespective of calendar time.

Upon HIV diagnosis, eligible men are assigned an ART initiation date. The local time-varying ART eligibility criteria is incorporated in the model such that PLHIV with a CD4 cell count < 350 cells $\mu {L}^{-1}$ (1996–2013), < 500 cells $\mu {L}^{-1}$ (2013–2016), and all PLHIV (2016 onwards) are eligible for ART. The effectiveness of ART in reducing HIV transmission is assumed to be 80% for the first 3–6 months after initiation [34], and 90–100% thereafter [35, 36] as not all those on ART will achieve sustained viral suppression [37]. Men can discontinue and later reinitiate ART. Following ART discontinuation, PLHIV continue to progress through lower CD4 cell count categories.

Survey data regarding the frequency of condom use were used to estimate the per-partner and per-act probability of using condoms. The categorical survey responses (never, occasionally, often, and always) for each stratum (partnership type, age, sero-status, sexual partnering level, and time) were converted into a numerical equivalent and averaged. An all-or-nothing approach is implemented for men in casual partnerships, while regular partners may protect only a proportion of their sex acts with condoms. Condoms are assumed to be 91% efficacious in preventing HIV transmission [38].

PEP, which is assumed to be 80% efficacious in preventing HIV acquisition [39], became available for non-occupational use for gbMSM at the end of 2000. Given the low PEP uptake at that time [8], PEP was initiated in the model in 2001 and uptake rates increased linearly until 2017, in line with observed trends in clinical settings [8]. PEP eligibility is modelled according to local clinical protocols.

In 2013, Québec recommended PrEP and partly reimbursed it using public funds [8]. The PrEP eligibility criteria incorporated within the model is consistent with local guidelines [40] and includes post-initiation HIV testing. PrEP is assumed to be 86% effective in inhibiting HIV acquisition [41]. A detailed description of the PEP and PrEP model components is presented in the Additional file 1.

##### Model calibration

An approximate Bayesian Computation-Sequential Monte Carlo (ABC-SMC) algorithm was used to select the parameter sets that best reproduce the following outcomes: age-stratified HIV prevalence (2005, 2008, 2017–2019), ART coverage (2005, 2017–2019), the proportion who ever used PrEP (2017–2019), the PrEP coverage (2017–2019), the CD4 cell count at diagnosis (2013–2017), and the proportion of PLHIV with knowledge of their status (2005, 2008, 2017). Additional cross-validation was performed by comparing the model outputs with surveillance data on: HIV incidence, the proportion of PLHIV with knowledge of their status (2018), the proportion of PLHIV who used PrEP in the past six months (2017–2019), the proportion of PLHIV with viral load suppression (2017–2019), and the age-stratified CD4 cell count at diagnosis (2013–2017) [42] and number of new diagnoses (2002–2017) [42]. The model calibration resulted in 100 posterior parameter sets. A full description of the calibration process can be found in the Additional file 1*.*

The model was initialized in 1975 and progressed in two-week increments. A total of 10,000 agents are simulated and results are scaled-up to the estimated population size of gbMSM in Montréal. Model simulations were run once for each posterior parameter set. The model was coded in R, version 3.6.3 [43], and incorporated C++ through the Rcpp interface, version 1.0.4.6 [44].

##### Model analysis

###### Description of the HIV epidemic

The estimated epidemic trajectories are described in terms of annual prevalence, incidence, and mortality rates (1975–2019). To identify the groups most affected by HIV, each measure is stratified by age group (15–24, 25–34, 35–44, 45–54, and 55 + years) and sexual partnering level (low, medium, and high).

###### Drivers of the HIV epidemic

Given the paucity of empirical behavioural data prior to 2000, the drivers of the epidemic are derived from that year onwards. Model outcomes are calculated from the beginning of the respective calendar year until the end of the year. We examined both who acquired and who transmitted infections. To characterize who acquired infections, we divided the number of men with a given characteristic (e.g., age, sexual partnering level) who acquired HIV over a defined period by the cumulative number of new HIV infections within that time period.

The proportion of incident infections transmitted was computed by dividing the cumulative number of transmission events from PLHIV with a given attribute (i.e., age, sexual partnership level, infection stage, knowledge of HIV status, ART status) over a defined time period by the cumulative number of new infections within that time period.

The mean and 90% credible interval (CrI), calculated as the 5^th^ and 95^th^ percentiles, of the 100 simulations are reported for each measure.

## Results

### Model calibration

The calibrated model adequately replicated the available age-specific epidemiological outcomes and intervention coverage data (Additional file 1). Specifically, the proportion diagnosed with HIV, the proportion virally suppressed, as well as the number of new diagnoses (stratified by CD4 cell count and age from 2013 onwards) provide credence in the model’s ability to capture population-level HIV transmission dynamics. While the modeled prevalence showed excellent agreement with that of the age-stratified survey data, the 2017 prevalence estimates for the 50 + year olds was underestimated for that year but remained in line with those from earlier surveys.

### Description of the epidemic

**Fig. 1:**
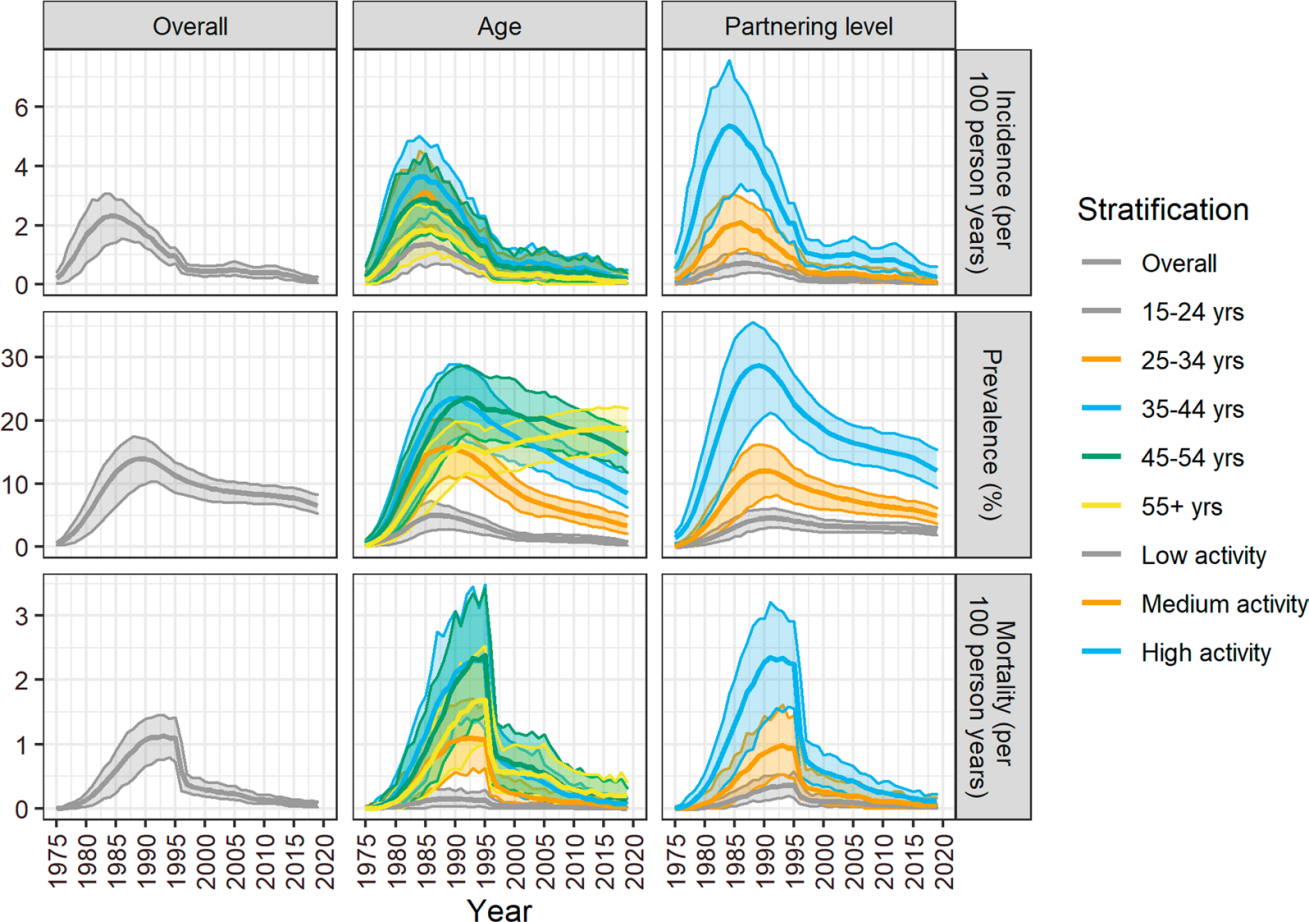
Overall, age, and sexual partnering level stratified estimates of HIV incidence, prevalence, and HIV-related mortality among men who have sex with men in Montréal over 1975–2019. The solid line represents the mean of 100 simulations, and the shaded area represents the 90% credible interval. The HIV incidence peaked in 1985, while the prevalence and mortality peaked in 1990 and 1993, respectively. The 35–44 year age group and high sexual partnering group had the highest prevalence, incidence and mortality at the epidemic peak, while the 45–54 year age group had the highest mortality. By 2017, the incidence was still highest within the 35–44 year age group. Subsequent to 1990, the HIV prevalence decreased for all age groups, with the exception of the 55 + year age group for which the prevalence continues to increase

The simulated HIV incidence peaked in 1985 (2.3 per 100 person years (PY), 90% CrI: 1.4–2.9 per 100 PY) and decreased to 0.1% (90% CrI: 0.04–0.3 per 100 PY) in 2019 (Fig. 1). Throughout the epidemic, the incidence among the 35–44 year age group was 2–3 times that of the 15–24 year age group. Additionally, the incidence ratio between the high and low sexual partnering groups ranged from 10 to 13 between 1975 and 1980, 8–9 between 1981 and 1985, and 5–7 thereafter, while the incidence ratio between the high and medium sexual partnering groups ranged between 2 and 4 throughout the epidemic.

The annual HIV prevalence peaked circa 1990 at 14% (90% CrI: 10–17%) (Fig. 1). During this time, the prevalence was highest among men aged 35–44 years (24%), and those in the high sexual partnering group (29%). Following this 1990 peak, the prevalence decreased for all strata, excluding men aged 55 + years who continue to experienced an increase in prevalence. By 2019, the overall prevalence had decreased to 7% (90% CrI: 5–8%), and the oldest age group and high sexual partnering group had the highest prevalence at 19 and 12%.

HIV-related mortality rates peaked in 1993 at 1.1 per 100 PY (90% CrI: 0.8–1. 5 per 100 PY). The mortality rate was highest among the 45–54 year age group, up to 10 times that of the 15–24 year olds from 1975 to 1988 and 11–33 times greater thereafter. From 1978 to 1983, the death rate among men in the high sexual partnering group was 10–26 times greater than that of the low sexual partnering group, and 4–9 times greater from 1984 onwards. The HIV-related mortality rate among the high sexual partnering group was 2–4 times that of the medium sexual partnering group from 1978 onwards.

### Drivers of the HIV epidemic

**Fig. 2:**
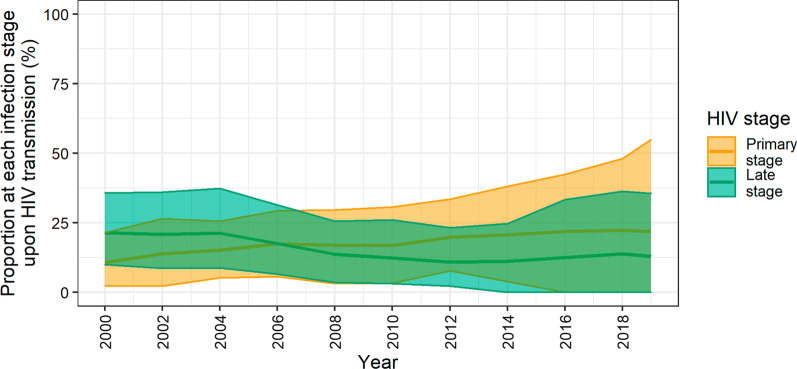
Proportion of annual HIV transmission events from individuals in the primary and late stages of infection. The mean (solid line) and 90% credible interval (shaded region) of 100 simulations are shown. The proportion of infections from men at the primary stage of HIV infection increased over time from a mean of 11% in 2000 to 22% in 2019. In contrast, men at the late stage of infection played a decreasing role in new infections

Over 2000–2019, the proportion of transmissions attributed to the primary stage of HIV infection increased from 11% (90% CrI: 2–21%) to 22% (90% CrI: 0–55%) (Fig. 2). The proportion of transmissions attributed to the late stage of disease decreased from 21% (90% CrI: 10–36%) in 2000 to 13% (90% CrI: 0–36%) in 2019.

**Fig. 3:**
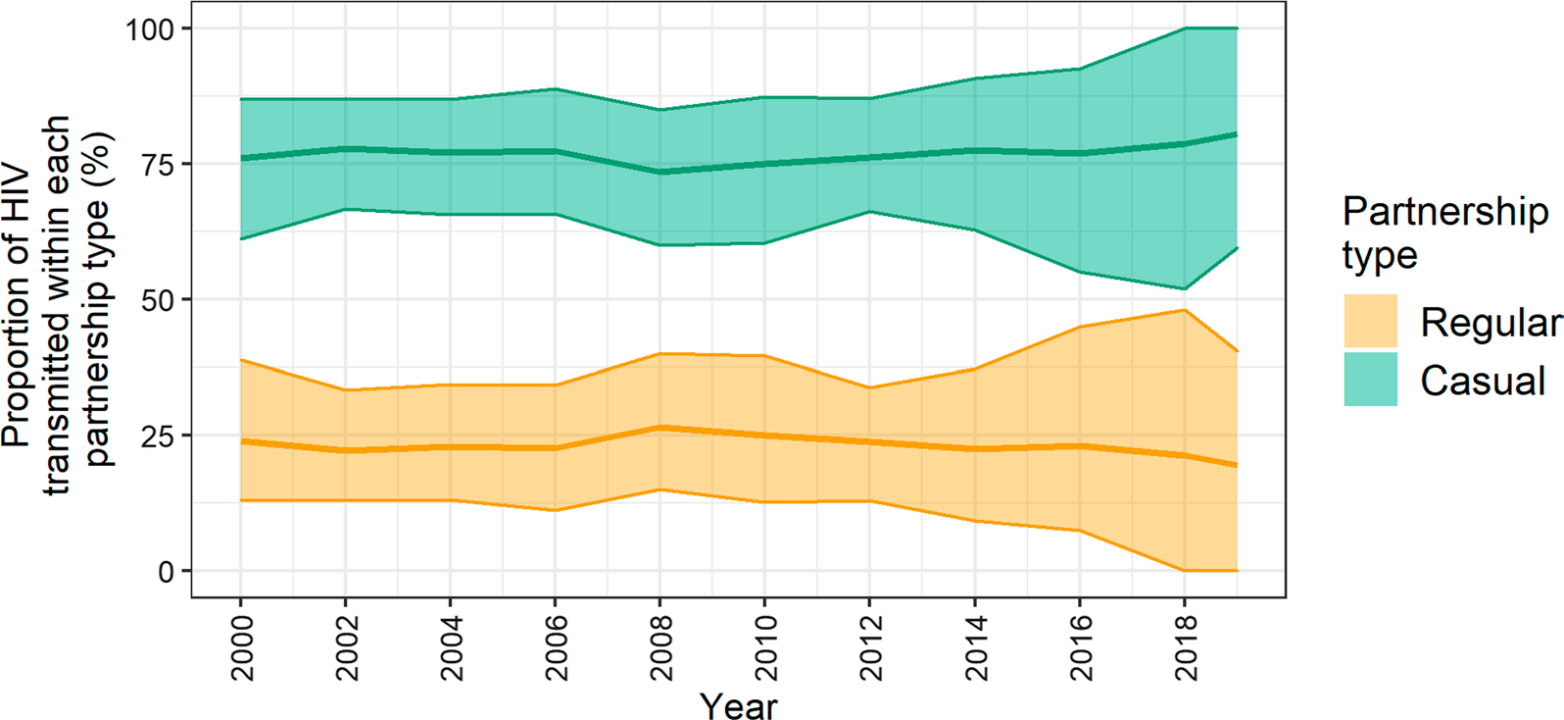
Proportion of annual incident HIV infections transmitted among casual and regular partners. Regular partnerships were defined as having a duration ≥ 4 weeks (two time steps). The mean proportion of transmissions (solid line) among each partnership type and the 90% credible interval (shaded region) are shown. Approximately 25% of new infections were transmitted within regular partnerships, while 75% of new infections were transmitted within casual partnerships

**Fig. 4:**
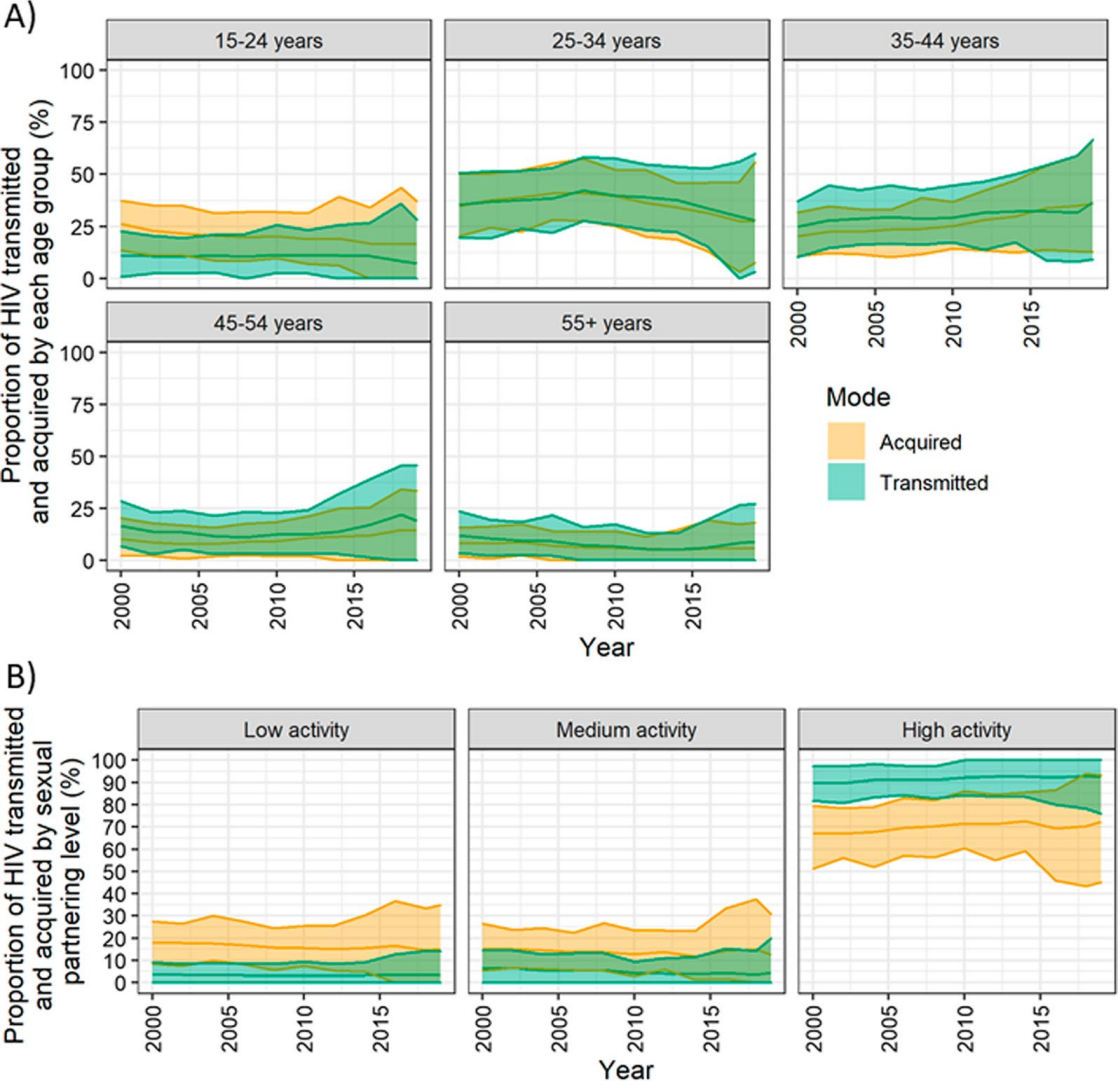
Mean and 90% credible interval of annual proportion of new infections transmitted and acquired by people living with HIV (PLHIV) stratified by age group (**A**) and sexual partnering level (**B**). Most new infections were transmitted and acquired by PLHIV aged 25–34 years between 1990 and 2017, and men aged 35–44 thereafter. With respect to sexual partnering level, through the epidemic, the majority of infections were transmitted by those with more than 10 partners per year

Within a given year, 19–26% and 74–81% of transmission events occurred within regular and casual partnerships, respectively (Fig. 3). Regarding age, the majority of infections were transmitted and acquired by individuals aged 25–34 years from 2000 to 2017, and those aged 35–44 thereafter (Fig. 4a). HIV transmissions and acquisitions among men aged 25–34 years peaked in 2008 and 2006, respectively, and decreased until 2019. Over 2000–2019, the 35–44 year age group played an increasing role in HIV transmissions (25–37%), and acquisitions (20–36%). Finally, the high partnering group transmitted (90–93%) and acquired (67–73%) most new infections throughout the epidemic. Of note, while the low/medium partnering groups each transmitted < 7% of infections, they acquired up to 18% of new HIV infections (Fig. 4b).

**Fig. 5:**
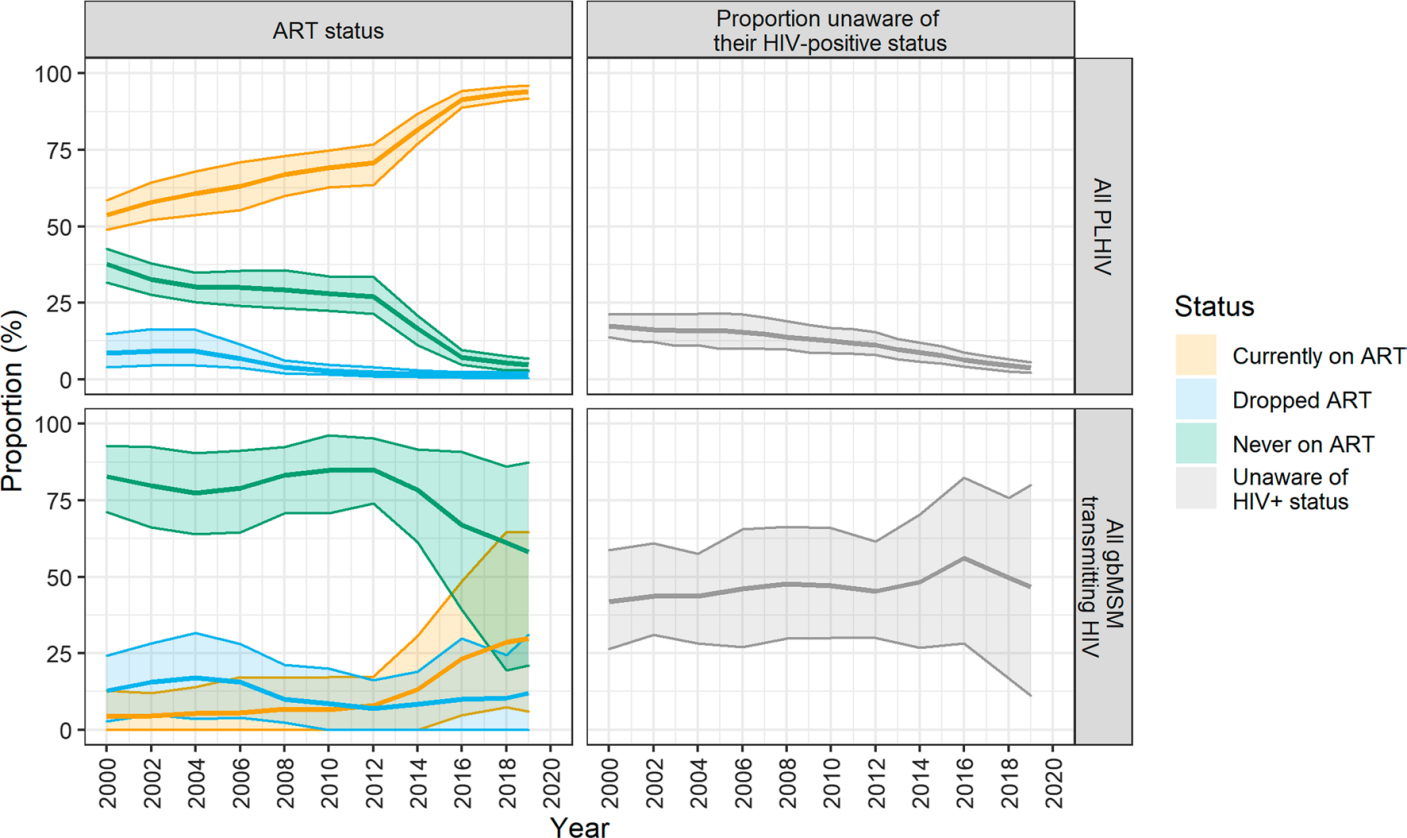
Proportion of people living with HIV (PLHIV) and those transmitting HIV who were unaware of their HIV-positive status, and their antiretroviral therapy (ART) status. The solid line represents the mean of 100 simulations and the shaded band represents the 90% credible interval. Over 2000–2019, the proportion of PLHIV unaware of their status decreased from 17 to 4%, while the equivalent proportion among those transmitting HIV varied between 41 and 56%. The proportion of PLHIV who had never been on ART decreased from 38 to 5%, while the proportion who had never been on ART at the time of transmitting HIV decreased from 85 to 58%

Between 2000 and 2019, the proportion of gbMSM unaware of their HIV status decreased from 17% (90% CrI: 14–21%) to 4% (90% CrI: 2–6%). The proportion who had never initiated ART decreased from 38% (90% CrI: 32–43%) to 5% (90% CrI: 3–7%), while the proportion who had discontinued ART for any length of time decreased from 9% (90% CrI: 4–15%) to 1% (90% CrI: 0–2%). During the same period, the proportion of transmission events from those unaware of their HIV-positive status varied between 42% (90% CrI: 27–59%) and 56% (90% CrI: 28–82%). The majority of transmission events occurred among PLHIV who had never been on ART. This proportion dropped from 85 to 58%, while the proportion of transmission events among men who had either discontinued ART or were on treatment (without a suppressed viral load) varied between 7–17% and 4–30% (Fig. 5).

## Discussion

Achieving city-level HIV elimination requires a granular understanding of local past and present transmission dynamics, the identification of gaps in the HIV care continuum, and data-driven implementation of appropriate interventions among the populations most impacted by HIV [45]. Through the development and analysis of a detailed model of HIV dynamics among gbMSM in Montréal, we characterized trends in HIV incidence, prevalence, and mortality, as well as the individual characteristics of HIV acquisitions and transmissions. Our model points to important improvements in epidemiological and continuum of care outcomes, with low HIV incidence in recent years.

At the beginning of the epidemic, the estimated rising mortality rates between 1985 and 1995, combined with some saturation of groups with higher partnering levels, could have played an important role in reducing onward HIV transmission. The latter translated into a decreasing HIV incidence and subsequent fall in prevalence over 1990–2000. The introduction of ART in 1996 is estimated to have led to a sharp drop in mortality, consistent with national estimates of deaths due to AIDS [1]. With the advent of these life-saving treatments and concomitant incidence reductions, the AIDS epidemic aged [46]. This was particularly salient with the increasing HIV prevalence among men aged 55 + years. This trend is similar to the national one in Canada where, despite an overall increase in the number of PLHIV, the number of gbMSM PLHIV has stabilized. Nationally, other groups of individuals such as people who inject drugs, Indigenous, and other heterosexual males and females contribute to the increasing national prevalence [48].

The large incidence ratio between the high and low sexual partnering groups is indicative of the underlying mixing patterns within the population and the increased likelihood of HIV transmission and acquisition among individuals with more sexual partners [49]. Consequently, the high partnering group experienced a greater reduction in incidence after the introduction of ART. The low/medium sexual partnering groups acquired approximately 20% of new HIV infections but transmitted few. This highlights their smaller role in ongoing transmission of HIV but suggests that renewed HIV prevention efforts that engage men in the low/medium sexual partnering groups are needed to further reduce HIV acquisition. Improving PrEP use among eligible men (e.g., those not using condoms) in these lower sexual activity groups could address such prevention needs. In addition, the model simulations indicate that the majority of transmission and acquisition events took place among gbMSM aged 25–44 years. This result is in line with local HIV surveillance data of new HIV diagnoses [3].

Our analyses identified opportunities to strengthen HIV prevention. Although we evaluate that diagnosis coverage has achieved high levels (> 90%) in recent years, approximately half of HIV transmission events in 2019 could have occurred from undiagnosed men. With the current low HIV incidence and high HIV testing rates, this undiagnosed fraction has considerably decreased over the last decade. ART coverage among gbMSM in Montréal was also estimated to be high in 2019 (> 90%). However, not all men on ART have a suppressed viral load and our model suggests that close to 1 out of 4 transmission events could be from PLHIV on treatment. There is a short 3–6 month period following ART initiation where viral load suppression has not been achieved and clinical data of gbMSM in Montréal suggest that 93% of those on ART are virally suppressed [37]. Reflecting this setting of low HIV incidence, high ART coverage, and low AIDS incidence, our model suggests that those in the acute infection stage have contributed a relatively larger proportion of transmission events over the last decade. These estimates are comparable with estimates found among gbMSM in the UK (population attributable fraction: 3–28% in 2014–15) [11] and Baltimore (8–35% over 2008–17) [14]. Given the heightened HIV transmissibility during the primary stage of infection [50], identifying new clusters of infections through the routine use of recency assays [51], phylogenetic testing [52], and genetic sequencing [53] could help achieve HIV elimination. Additionally, continued efforts are necessary to increase timely diagnosis, prompt linkage to ART, the continued promotion of “U = U” (i.e., undetectable equals untransmittable) [54] to reduce stigma, and sustaining viral load suppression among those who have achieved it.

The study results should be interpreted considering several limitations. First, the paucity of recorded empirical data from the early years of the epidemic (e.g., condom use, sexual behaviors, and HIV testing) necessitated the use of informed assumptions for sexual behaviors. However, cross-validation of model outputs with surveillance data on new HIV diagnoses from 2002 to 2017, suggest that epidemic trends are accurately reflected. Second, the model does not explicitly model HIV transmission through injecting drug use among gbMSM but provincial surveillance data suggest few HIV diagnoses in this combined category. Third, the survey instruments had different eligibility criteria and, in some cases, metrics had slightly different definitions across surveys. However, statistical methods were used to standardize the survey data, ensuring their comparability and improving representativeness. Fourth, ethnicity was not incorporated in the model but studies suggest that in Canada, there are minimal differences in HIV prevalence by race [55]. Nevertheless, we recognize that some minoritized populations could face distinct barriers to HIV prevention. Finally, the lower HIV incidence in recent years resulted in large uncertainty when performing stratified data analyses.

## Conclusions

HIV incidence among Montreal gbMSM has decreased to low levels in recent years. To achieve the goal of HIV elimination, continued surveillance efforts and the strategic implementation of combination HIV prevention strategies, including increased frequency of HIV testing among high sexual partnering individuals. By characterizing the HIV dynamics, it was possible to identify the population subgroups most impacted by HIV and the need to further improve ART retention. The implementation of interventions that focus on gbMSM aged 25–45 years, and gbMSM in the high partnering group could help to achieve HIV elimination among gbMSM in Montréal.

## Supplementary Information

**Additional file 1.** Additional materials.

## Data Availability

The data that support the findings of this study are available from Engage-Montréal but restrictions apply to the availability of these data, which were used under license for the current study, and so are not publicly available. Data are however available from the authors upon reasonable request and with permission of Engage-Montréal.

## Abbreviations

ABM: Agent-based model
ART: Anti retroviral therapy
CrI: Credible interval
gbMSM: Gay, bisexual, and other men who have sex with men
PEP: Post-exposure prophylaxis
PLHIV: People living with HIV
PrEP: Pre-exposure prophylaxis
PY: Person years
